# Screening, Cloning, Expression and Characterization of New Alkaline Trehalose Synthase from *Pseudomonas monteilii* and Its Application for Trehalose Production

**DOI:** 10.4014/jmb.2106.06032

**Published:** 2021-08-20

**Authors:** Srisakul Trakarnpaiboon, Benjarat Bunterngsook, Rungtiva Wansuksriand, Verawat Champreda

**Affiliations:** 1 Enzyme Technology Research Team, Biorefinery and Bioproduct Technology Research Group, National Center for Genetic Engineering and Biotechnology, 113 Thailand Science Park, Paholyothin RD., Klong Luang District, Pathumthani 12120, Thailand; 2 Cassava and Starch Technology Research Team, Functional Ingredients and Food Innovation Research Group, National Center for Genetic Engineering and Biotechnology, Bangkok 10900, Thailand

**Keywords:** Trehalose, trehalose synthase, maltose, *Pseudomonas monteilii*

## Abstract

Trehalose is a non-reducing disaccharide in increasing demand for applications in food, nutraceutical, and pharmaceutical industries. Single-step trehalose production by trehalose synthase (TreS) using maltose as a starting material is a promising alternative process for industrial application due to its simplicity and cost advantage. *Pseudomonas monteilii* TBRC 1196 was identified using the developed screening method as a potent strain for TreS production. The TreS gene from *P. monteilii* TBRC 1196 was first cloned and expressed in *Escherichia coli*. Purified recombinant trehalose synthase (PmTreS) had a molecular weight of 76 kDa and showed optimal pH and temperature at 9.0 and 40°C, respectively. The enzyme exhibited >90% residual activity under mesophilic condition under a broad pH range of 7-10 for 6 h. Maximum trehalose yield by PmTreS was 68.1% with low yield of glucose (4%) as a byproduct under optimal conditions, equivalent to productivity of 4.5 g/l/h using enzyme loading of 2 mg/g substrate and high concentration maltose solution (100 g/l) in a lab-scale bioreactor. The enzyme represents a potent biocatalyst for energy-saving trehalose production with potential for inhibiting microbial contamination by alkaline condition.

## Introduction

Trehalose or α-D-glucopyranosyl-α-D-glucopyranoside is a non-reducing disaccharide certified by the US Food and Drug Administration as a Generally Recognized as Safe (GRAS) ingredient in 2000 [1]. It has various applications in the food, cosmetic and pharmaceutical industries as a preservative, stabilizer or moisturizer for frozen foods, vaccines and skincare products [2-4]. Trehalose can be produced by chemical and biosynthesis. Chemical synthesis utilizes the reaction of 2,3,4,5-tetra-O-acetyl-D-glucose with 3,4,6-tri-O-acetyl-1,2-anhydro-D-glucose in benzene but with a low yield and at high cost [1, 5]. In the biological route, trehalose is produced as a source of energy, and functions in cell protection under stress condition in living organisms [6-8].

Currently, five different biosynthesis pathways have been reported for trehalose production in microbes as (1) trehalose-6-phosphate synthase and trehalose-phosphatase (TPP), (2) maltooligosyltrehalose synthase and maltooligosyltrehalose trehalohydrolase (MTHase/MTHase), (3) trehalose synthase (TreS), (4) trehalose phosphorylase (TreP), and (5) trehalose glycosyltransferring synthase (TreT) [1, 2]. The major disadvantage in the application of the three pathways (TPP, TreP, and TretT) is mainly in the need to use glucose and derivatives of glucose (glucose-6-phosphate, ADP-glucose or glucose-1- phosphate) as the starting materials [2, 9]. This makes up-scaling of the processes for industrial application economically infeasible due to the cost of preparing the glucose derivatives. The MTHase/MTHase gave high trehalose yield and has been implemented for commercial production of trehalose from starch [10-13]; however, generation of mixed byproducts (branched starch, maltose and maltotriose) and the long reaction time needed for the two-step enzymatic reaction are disadvantageous [13]. Hence, an alternative trehalose production process by TreS has attracted research interest.

A one-step trehalose production from maltose can be catalyzed by TreS. TreS enzymes are found in several bacteria and archaea such as *Thermus aquaticus*, *Mycobacterium smegmatis*, *Picrophilus torridus*, *Deinococcus geothermalis*, *Corynebacterium glutamicum* and *Pseudomonas stutzeri* [14-19]. This enzyme catalyzes an intramolecular rearrangement of the α-1,4-glycosidic bond of maltose to the α,α,-1,1-linkage of trehalose in the absence of coenzyme [20], making it a potential biocatalyst for simple conversion of maltose to trehalose. TreS enzymes have been characterized from many microorganisms but most have low stability under operational conditions and generate high glucose as a byproduct [13].

Production of maltose syrup from cassava is a well-established industry in Thailand. Valorization of the low-priced maltose to secondary products of higher commercial value is of great interest for the industry. Our aim in this study is to discover a new TreS enzyme with properties desirable for industrial application, *i.e.*, high stability under operating conditions with low generation of glucose as a byproduct. A protocol for screening TreS-producing strains was developed with the use of effective induction media. The TreS gene from a potential strain was cloned and expressed and the recombinant TreS was characterized for its biochemical properties. The alkaliphilic property of the enzyme is considered advantageous as it inhibits contaminated microbes while enabling operation under energy saving mesophilic conditions. Conversion of maltose to trehalose using this enzyme was investigated in a bioreactor, showing its potential for future biotechnological application.

## Materials and Methods

### Bacterial Strains, Plasmids and Culture Conditions

Microorganisms for screening and selection for TreS production were obtained from the Thailand Bioresource Research Center (TBRC) (www.tbrcnetwork.org) and the Thailand Institute of Scientific and Technological Research (TISTR) (https://www.tistr.or.th/tistr_culture). *P. monteilii* TBRC 1196 from the Thailand Biological Resource Center was used as a source of the TreS gene. The bacteria were cultivated in nutrient broth medium on a rotary shaker at 30°C. *E. coli* DH5α and BL21 (DE3) (Novagen, USA) were used as a cloning host and expression host, and cells with plasmids were cultured at 37°C in Luria-Bertani broth supplemented with ampicillin (34 μg/ml) and kanamycin (50 μg/ml), respectively.

### Method for Screening of TreS-Producing Strains

The bacteria were cultivated in three screening media. The first was the growth medium recommended for each strain by TBRC and TISTR (nutrient broth, marine agar, MRS broth, or ISP2 broth). The second was the osmotic medium prepared by addition of 1% (w/v) NaCl to the growth media [21], and the third was maltose medium consisting of 10% (w/v) maltose, 5% (w/v) peptone, 1% (w/v) yeast extract, 1% (w/v) K_2_HPO_4_, 1% (w/v) NaH_2_PO_4_, 0.5% (w/v) MgSO_4_·7H_2_O and 0.1% (w/v) CaCl_2_ as modified from Zhu *et al*. with an addition of maltose [22]. All target strains were grown in 5 ml cultures at 30°C with agitation at 200 rpm for 24 and 48 h. The cells were pelleted by centrifugation at 6,000 ×*g* for 10 min at 4°C and kept at -20°C overnight. Then, the cells were resuspended in 1 ml of 50 mM potassium phosphate buffer (pH 7.0). The cells were lysed using an Ultrasonic Processor VCX 130 PB (Sonics & Materials, Inc., USA) with a 3 mm diameter probe at a frequency of 20 kHz and 80% of amplitude for 30 and 60 sec. Crude cell extracts were analyzed for protein content and TreS activity.

The TreS activity was assayed based on trehalose released from maltose according to the method modified from Cai *et al*. [23]. To validate the rapid trehalose assay protocol for screening, trehalose standard solution (2, 4, 10, 20, 60, and 100 g/l) was analyzed by a Trehalose Assay Kit (Megazyme International, Ireland) and high-performance liquid chromatography (SPD-M10A DAD, Shimadzu, Japan) equipped with a refractive index detector and a Shodex HILICpak VG-50 4E column (4.6 × 250 mm, 5 μm) at 1.0 ml/min. Column oven temperature was set at 40°C and the mobile phase consisted of a mixture of 80% acetonitrile and 20% ultrapure water.

For TreS activity assay, 0.5 ml of the crude cell extracts was mixed with 0.5 ml of 5% (w/v) maltose solution in 50 mM potassium phosphate buffer (pH 7.0). The reaction mixture was incubated at 40°C for 3 h. The reaction was stopped by heating at 95°C for 15 min, and the mixture was centrifuged at 4°C and 8,000 rpm for 5 min. The amount of trehalose in the reaction mixture was measured by a Trehalose Assay Kit (Megazyme). One TreS unit is the amount of enzyme required to produce 1 μmol/min of trehalose from maltose.

### Cloning and Expression of TreS Gene

The DNA of *P. monteilii* TBRC 1196 was prepared using a bacterial genome DNA extraction kit (Thermo Scientific, Inc., USA). The putative gene encoding TreS was amplified by a polymerase chain reaction (PCR) using PmTreS forward (5′-GCCCATGGCG**ATG**ACCCAACCCGACCCGTCATAC-3′) and reverse (5′-CTCGAG**GAC**ATGCCCACTGCTGTTGACGAT-3′) primers designed based on the TreS gene from *P. monteilii* SB3101. The recognition sites for *Nco*I and *Xho*I are underlined and the start and stop codons are in bold letters [24]. The PCR reactions contained DNA template, 10 mM dNTPs, 10 μM primers, DNA polymerase, 10x GC buffer and nanopure water. The target gene was amplified using the following conditions: pre-denaturation (98°C, 30 sec), 30 cycles of denaturation (98°C, 10 sec), annealing (64°C, 30 sec), extension (72°C, 2 min) and prolonged elongation (72°C, 10 min). The PmTreS gene from the PCR reaction was purified using a GeneJET Gel Extraction Kit (Thermo Scientific, Inc.), then cloned into a pJET1.2 vector (Fermentas UAB, Lithuania) and transformed into *E. coli* DH5α for sequencing. The purified pJET1.2 vector containing the PmTreS gene fragment was digested with *Nco*I and *Xho*I, ligated into pET-28a(+) (Novagen) digested with the same restriction enzymes and transformed into *E. coli* BL21(DE3) for enzyme expression.

Sequence analysis of PmTreS was performed on the BLAST server of the National Center for Biotechnology Information (https://blast.ncbi.nlm.nih.gov/Blast.cgi). Multiple amino acid sequence alignment was performed using the online program Clustal Omega (https://www.ebi.ac.uk/Tools/msa/clustalo/).

The *E. coli* BL21 (DE3) cells harboring the pET28a-PmTreS plasmid were cultured in Luria-Bertani broth supplemented with 50 μg/ml of kanamycin and incubated at 37°C. When the optical density of the culture reached 0.6, IPTG was added to a final concentration of 1 mM. Then, PmTreS was further cultivated at 37, 25, and 18°C for 3, 6, and 20 h, respectively.

### Purification of Recombinant TreS

The culture broth was centrifuged at 6,000 ×*g* for 10 min and the pellet was re-suspended in 10 mM potassium phosphate buffer (pH 7.0). The cells were disrupted by sonication as described above. The cell extract was clarified by centrifugation at 6,000 ×*g* for 30 min and applied to a His trap FF column (GE Healthcare, UK), pre-equilibrated with the same buffer. Unbound proteins were removed by washing with the same buffer. Bound proteins were eluted with the elution buffer (20 mM potassium phosphate buffer, pH 7.4, and 500 mM NaCl, and 60 mM imidazole). Protein concentration was determined using a Bio-Rad Protein Assay Kit (BioRad Laboratory, USA). The protein profiles in collected fractions were analyzed by sodium dodecyl sulfate polyacrylamide gel electrophoresis (SDS-PAGE).

### Biochemical Characterization of TreS

The effect of temperature on TreS activity was measured at 20-70°C in 50 mM potassium phosphate buffer (pH 7.0). For optimal pH determination, enzyme activity at various pH values was examined at 40°C in a buffer containing 1% maltose. The following buffers were used: 50 mM sodium acetate (pH 3.0-5.0), 50 mM potassium phosphate (pH 6.0-8.0), and 50 mM glycine-NaOH (pH 9.0-11.0).

Thermal stability was determined by measuring residual enzyme activity after pre-incubation of the enzyme without the substrate at 20 to 70°C for 1, 2, 4, 6, 12, and 24 h. To determine the effect of pH on stability, the recombinant enzyme was first incubated in buffers with different pH values ranging from 3.0 to 7.0. After incubation at 30°C for 24 h, the residual enzyme activity was measured at 40°C and pH 7.0 using the method described above.

The effects of metal ions (Zn^2+^, Mg^2+^, Ca^2+^, Cu^2+^, Ba^2+^, Ni^2+^, and Fe^2+^) on enzyme activity were assayed at a concentration of 1 mM. All metals were used in chloride form. The enzyme was pre-incubated with 3 mM EDTA in 50 mM potassium phosphate buffer, pH 7.0, for 30 min at 30°C. The excess EDTA was removed by buffer exchange using centrifugation tubes (Mw cut-off 10 kDa). The amount of trehalose was determined by HPLC as described above. Relative activity without any metal ions was defined as 100%.

To determine the effects of substrate concentration on enzyme activity, the purified enzyme was mixed with 50 mM glycine-NaOH buffer (pH 9.0) containing various maltose concentrations of 10, 50, 100, 150, 200, and 250 g/l and incubated at 40°C for 24 h.

### Trehalose Production from Maltose in Bioreactor

Production of trehalose was preliminarily studied using purified PmTreS (2 mg/g of substrate) in a reaction containing 50 ml of 100 g/l maltose in 50 mM potassium phosphate buffer pH 7.0, 8.0 and 50 mM glycine-NaOH pH 9.0, and incubated at 30 and 40°C for 24 h. Up-scaled trehalose production by TreS was carried out in a 2-L stirred tank bioreactor (MDET-N-2L, B.E. Thailand) using 1 L of maltose (100 and 200 g/l) as a substrate, at 40°C and pH 9.0 with mixing at 50 rpm under the same condition as described above. Samples were taken at different time intervals and heated at 100°C for 10 min to stop the reaction. The trehalose produced was measured by HPLC.

### Analysis

Sugar assay was performed following the method of Kuschel *et al*. [25] with minor modifications. Concentrations (%, w/v) of glucose, maltose and trehalose were analyzed on a high-performance liquid chromatograph (LC-20ASeries, Shimadzu-GL Sciences, Japan) equipped with a Shodex HILICpak VG-50 4E column (4.6 × 250 mm, 5 μm) column at 40°C. Acetonitrile:ultrapure water (80:20 v/v) was used as the mobile phase at a flow rate of 1.0 ml/min.

The total amount of protein in crude cell extracts was analyzed by the Bradford method using a Bio-Rad Protein Assay Kit (BioRad Laboratory) with bovine serum albumin (BSA) as a standard.

### Statistical Methods

The data obtained were subjected to one-way analysis of variance using SPSS statistical software, release 11.5 (SPSS Inc., USA). Duncan’s multiple range test was performed to determine significant differences of the means, with significance level set at *p* ≤ 0.05.

## Results and Discussion

### Development of Protocol for TreS-Producing Strain Screening

The traditional method for screening TreS required a large volume of culture and preparation of crude cell extract and trehalose detection was time consuming [26, 27]. In this study, a modified screening method was developed by selection of screening media for small-scale cultivation, optimization of the crude extract preparation method and validation of enzyme activity assay on a small scale. First, the three screening media, including the optimal growth medium, optimal growth medium plus NaCl (salt stress condition) and maltose medium (sugar stress condition) were investigated to select the potential medium for screening on a small scale (5 ml in screw-cap culture tubes). Twenty-one strains from 14 genera previously reported for TreS production were investigated as the candidate strains in this experiment. The results showed that the maltose medium was the most potent for screening of TreS-producing strains because 19 of 21 strains showed TreS activity when cultured in this medium for 24-48 h, whereas trehalose synthase activity from some strains could not be found in the other two media (Fig. S1A). Microorganisms produced an intracellular trehalose content to enable the cells to resist physicochemical stresses [28]. Addition of sugars and inorganic salts in the medium can create stress conditions leading to increased trehalose production. An increase in intracellular trehalose was reported to promote growth of the microbes under osmotic stress [21, 29]. Results showing different levels of TreS activity obtained from different media for the same strain might be due to diverse types of stresses and induction effects that resulted in variation in trehalose formation by each strain. However, some strains did not show TreS activity in all screening media, suggesting their inability to produce enzyme.

Subsequently, two methods including freeze-thawing at -20°C and sonication at 30 and 60 sec were investigated to reduce cell extraction time. The results revealed that protein contents in cell extraction obtained at 30 and 60 sec of sonication were similar (Fig. S1B), whereas the freeze-thaw method resulted in less protein content than the sonication method. Therefore, sonication at 30 sec was chosen for the rapid screening protocol.

To set up the high-throughput method for trehalose analysis in the screening protocol, the trehalose assay kit was applied and compared with the HPLC method. Data on trehalose concentration from these two methods were plotted to see their correlation. The results showed that the two methods correlated with good agreement in the range of 0-100 g/l of trehalose, suggesting that the trehalose assay kit could be used to estimate TreS in the screening process (Fig. S1C). The developed method is advantageous in terms of time, throughput, and reaction scale compared to previously reported methods, using the screening protocols based on TLC or HPLC [26, 27](Fig. S2).

The developed screening method was then applied for the screening experiment. Target microorganisms for screening were chosen from genera previously reported for TreS or maltose α-D-glucosyltransferase gene by TBRC and TISTR. Of 169 strains screened, 60 strains showed TreS activity (Fig. S3). Among these, *P. monteilii* TBRC 1196 was selected as it showed the highest ability to convert maltose into trehalose compared to the other strains. The enzyme from this identified bacterial strain was selected for further characterization.

### Identification, Cloning and Sequencing of TreS Gene from *P. monteilii*

The TreS gene from *P. monteilii* TBRC 1196 with a length of 2,091 bp was successfully amplified (see Fig. S4). The gene encoded a polypeptide containing 697 amino acid residues with a predicted molecular mass of 76.7 kDa. Amino acid sequence comparison of mesophilic TreS indicated that PmTreS showed the highest identity of 96% to PmTreS_SB3101_: *P. monteilii* [GenBank: AHC88446.1] and 95% identity with PmTreS_SB3078_: *P. monteilii* [GenBank: AHC83070.1] and TreS from *P. putida* [GenBank: QNG09137.1], 71% identity with that of *P. stutzeri* [GenBank: AAF26837] and 20-30% identity with PpTreS: *P. putida* [GenBank: TFF50727.1], AcTreS: *Arthrobacter chlorophenolicus* [GenBank: AKA87409.1], AaTreS: *A. aurescens* [GenBank: ACL80570.1], DrTreS: *D. radiodurans* [GenBank: AEJ36289], RoTreS: *Rhodococcus opacus* [GenBank: AGF84773.1], EhTreS: *Enterobacter hormaechei* [GenBank: ACI16355.1] and CgTreS: *C. glutamicum* [GenBank: CAF20645]. It should be noted that the TreS enzymes from *P. monteilii* SB3101 and SB3078 are only reported for their putative gene sequences in the genomes and their biochemical properties have not been characterized yet [24].

**Fig. 1:**
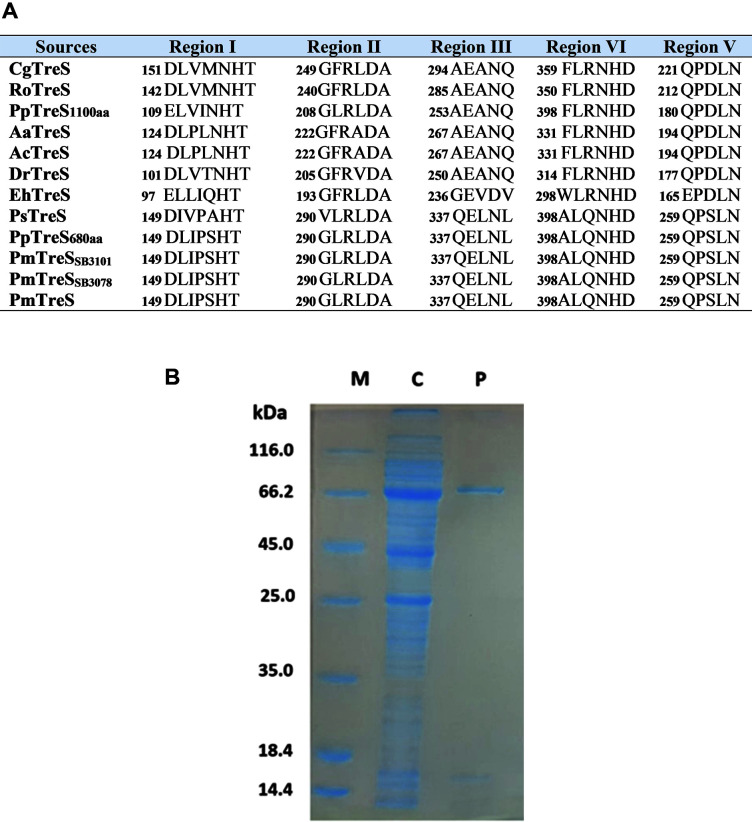
Comparison of amino acid sequence alignment at the conserved regions of TreS from mesophilic microorganisms (**A**) and SDS-PAGE analysis of PmTreS purification. (**B**) Lane C; crude extracts, Lane P; purified PmTreS from Ni Sepharose affinity chromatography column and Lane M; markers. PmTreS: *P. monteilii* TBRC 1196 (this study), PmTreS_SB3101_: *P. monteilii* [GenBank: AHC88446.1], PmTreS_SB3078_: *P. monteilii* [GenBank: AHC83070.1], PpTreS680aa: *P. putida* (680aa) [GenBank: QNG09137.1], PsTreS: *P. stutzeri* [GenBank: AAF26837], PpTreS: *P. putida* (1106aa) [GenBank: TFF50727.1], AcTreS: *A. chlorophenolicus* [GenBank: AKA87409.1], AaTreS: *A. aurescens* [GenBank: ACL80570.1], DrTreS: *D. radiodurans* [GenBank: AEJ36289], RoTreS. *R. opacus* [GenBank: AGF84773.1], EhTreS: *E. hormaechei* [GenBank: ACI16355.1], and CgTreS: *C. glutamicum* [GenBank: CAF20645].

Multiple sequence alignment showed the five conserved sequence motif regions of glycoside hydrolase family 13 (GH13) [30]. The five conserved regions including the seven highly conserved amino acid residues (His154 in region I, Arg292 and Asp294 in region II, Glu338 in region III, His402 and Asp 403 in region IV and Ser261 in region V) were also identified in the PmTreS sequence (Fig. 1A). The important roles of amino acid in the conserved region of TreS were studied in previous reports. Mechanistic analysis of trehalose synthase from *M. smegmatis* revealed that Asp230 is a catalytic nucleophilic residue, Glu272 is a general acid/base catalyst, and His341 and Asp342 are conserved carboxylic acids [31]. Furthermore, His120, Asp217, Glu259, Asp329 and His328 significantly affected the catalytic activity of TreS from *Thermomonospora curvata* DSM 43183 [32]. Moreover, the roles of the five conserved amino acids of TreS from *R. opacus* were explained. Asp244 and Asp354 might function as nucleophiles that attack the bonded anomeric carbon of maltose, while Glu286 might play a role in proton donation. His147 and His353 were suggested to act as substrate binding sites [33].

### Expression and Purification of PmTreS

The recombinant plasmid was transformed into the *E. coli* BL21(DE3) strain for expression. The results showed that induction with 1 mM IPTG at 18°C for 12 h was the optimal condition for expression of the recombinant protein in soluble form (Fig. S5). Higher temperature led to higher level formation of inclusion bodies, while lower IPTG concentrations decreased the total amount of recombinant protein in the soluble fraction. The apparent molecular weight of the purified PmTreS was approximately 76 kDa (Fig. 1B), which agreed well with the calculated Mw of 76.72 kDa. The size of TreS from our strain was comparable to the respective enzyme from *P. stutzeri* CJ38 [19].

### Effects of pH and Temperature on TreS Activity and Stability

**Fig. 2:**
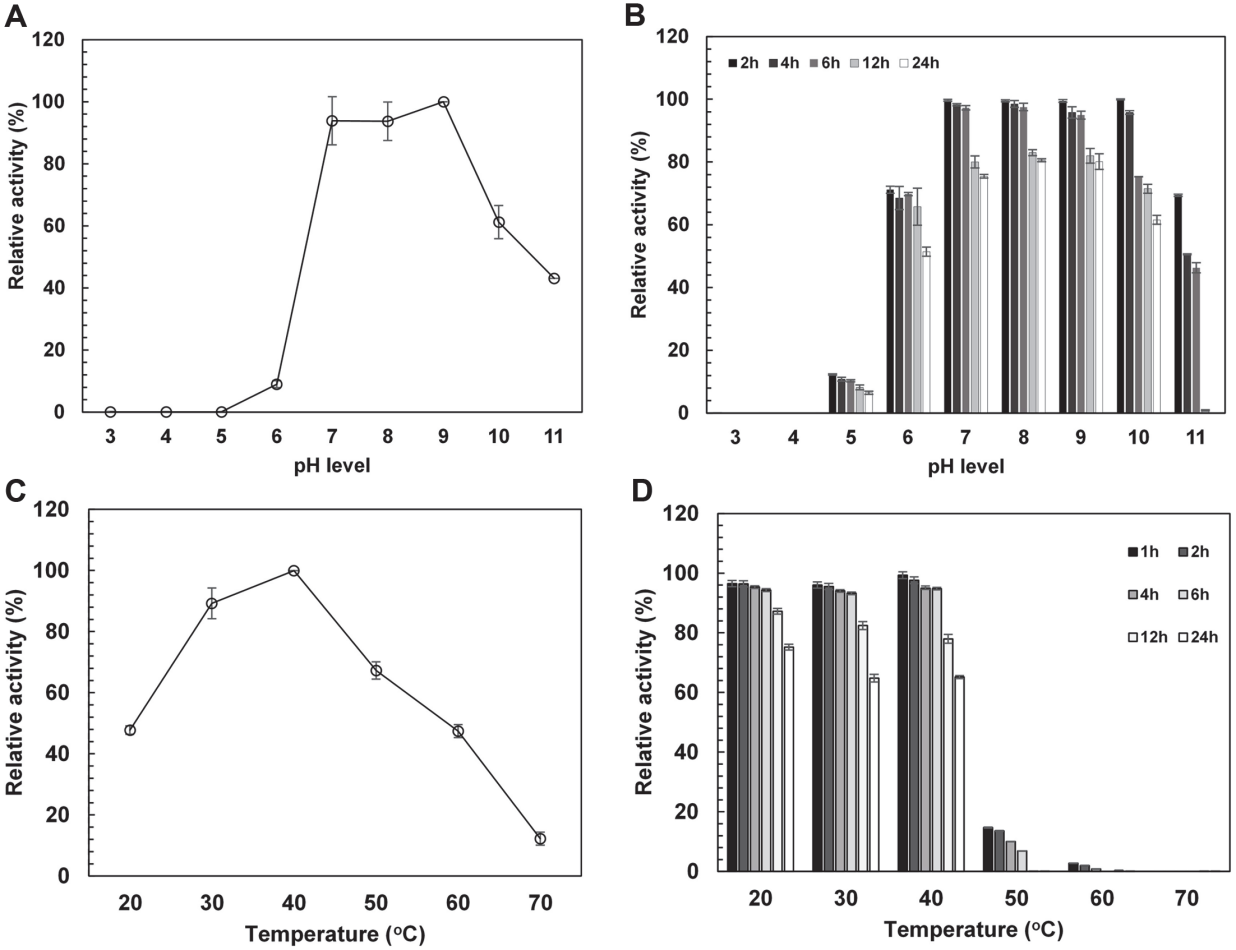
Effects of pH and temperature on PmTreS activity and stability. (**A**) Relative activity at different pHs, (**B**) stability at different pHs, (**C**) relative activity at different temperatures, (**D**) thermostability. For pH stability determination, the enzyme was pre-incubated at 30°C at different pHs before measuring activity at 40°C, pH 7.0. For thermostability test, the enzyme was pre-incubated at different temperatures at pH 7.0 before determining activity at 40°C, pH 7.0. One hundred percent of enzyme activity was 6.4 U/mg protein.

The effects of pH and temperature on PmTreS activity and stability are shown in Fig. 2. Optimal pH of the PmTreS was 9.0 (Fig. 2A). However, the enzyme maintained high activity at pH 7.0-9.0, with more than 80%remaining activity after pre-incubation for 12 h and 60% after 24 h (Fig. 2B). The pH stability of PmTreS was more stable under alkaline condition compared to other TreS enzymes reported in Table 1. Operation of reactions under alkaline condition is considered advantageous as this is unfavorable for the growth of many microorganisms. Most bacteria prefer a pH range of approximately 5–8 whereas most fungi grow optimally at a pH range of approximately 5.0–6.0 [34]. This suggests the effects of alkalinity on inhibition of microbial contamination.

The optimal temperature for this enzyme was 40°C (Fig. 2C), which was higher than that for many previously reported TreS enzymes isolated from mesophilic microorganisms as shown in Table 1, for example *Pseudomonas* sp. P8005, *P. stutzeri* CJ38, *P. putida* NBRI0987, *C. glutamicum* ATCC13032, *D. radiodurans* DSMZ 20539, *R. opacus* ACCC 41021, *A. aurescens* CGMCC 1.1892 and *A. chlorophenolicus* SK33.001, which showed optimal temperatures ranging from 25 to 37°C [17-19, 23, 33, 35-37] but lower than those for TreS enzymes from thermophilic microorganisms such as *Thermus* sp., *Thermobaculum* sp., *Picrophilus* sp. and *Meiothermus* sp., which have an optimal temperature range between 45-65°C [16, 22, 38-40]. All of the reported TreS enzymes from mesophilic and thermophilic sources showed their optimal pH in the neutral or less alkaline range compared to the PmTreS, except for the enzyme from *Thermus* sp., had an optimal pH at 9, similar to our enzyme. For the stability test, PmTreS showed more than 90% remaining activity for 6 h and almost 70% for 24 h at 20 to 40°C, while its activity markedly dropped to lower than 30% at 50°C, as shown in Fig. 2D. Its thermostability at up to 40°C was higher than that of many previously reported TreS enzymes from mesophilic microbial sources that showed only 80% activity remaining after incubation at 40°C for 30 min [13, 18, 35]. High activity and stability at the optimal mesophilic temperature allow cost saving in terms of required heating energy [13] and are considered desirable characteristics for its use in cell surface-displayed forms [9, 41, 42].

### Effects of Metal Ions and Inhibitors on Activity

The activity of PmTreS significantly increased (*p*-value ≤ 0.05) with addition of metal ions (Mg^2+^, Ca^2+^, and Mn^2+^) whereas the presence of Zn^2+^, Cu^2+^ and Ni^2+^ strongly inhibited enzyme activity (Table 2). It is well known that glycosidase activity is affected by the presence of metal ions [43]. Some cations like Ca^2+^ are required by other enzymes in the GH13 hydrolase family, which stabilizes the linkage of the (α/β)8-barrel [44, 45]. Inhibition of some GH13 hydrolases by Cu^2+^ could be due to competition between the exogenous cation and the protein-associated cations, resulting in decreased enzyme activity [46, 47]. The Cu^2+^ cation also showed a strong inhibitory effect on TreS from previously reported bacterial species such as *P. stutzeri*, *P. torridus*, *A. aurescens*, *E. hormaechei*, *C. glutamicum*, *M. ruber* and *D. radiodurans* [16-19, 22, 37 ,48]. However, the addition of metal ions such as Fe2+ and Ba2+ did not significantly affect activity (*p*-value > 0.05).

### Effects of Substrate Concentration and Enzyme Loading on Trehalose and Glucose Formation

**Fig. 3:**
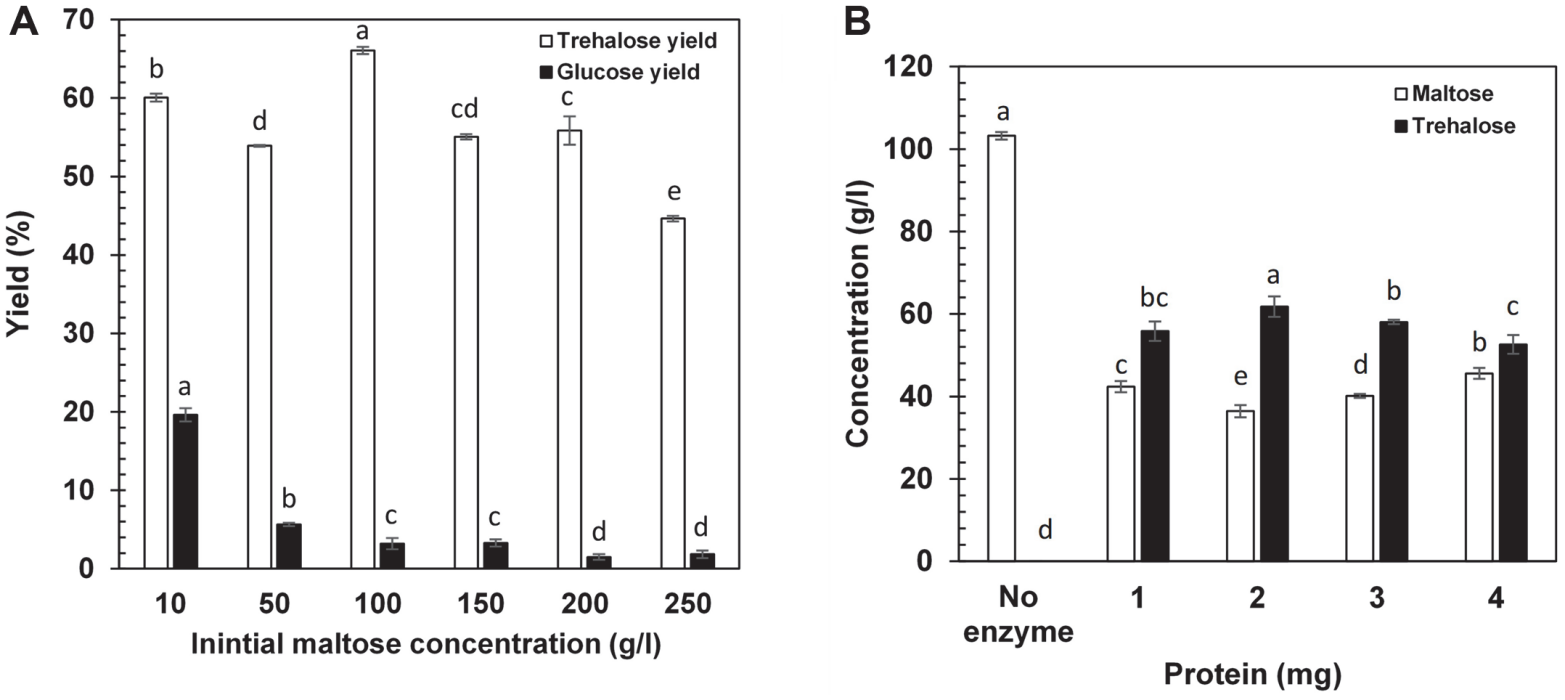
Effects of substrate concentration (**A**) and protein concentration (**B**) on trehalose production by PmTreS. The reactions were carried out in 50 mM glycine-NaOH buffer (pH 9.0) at 40°C for 24 h.

The effect of substrate concentration on yield and specificity of trehalose by PmTreS was examined at pH 9.0 and 40°C using TreS loading of 2 mg/g substrate. Yields of trehalose at maltose concentrations of 10, 50, 100, 150, 200, and 250 g/l were between 44.6 and 66.1%. Glucose yields were observed at 1.8-5.6%, except at 10% maltose concentration which showed a high glucose yield of 19.6% (Fig. 3A). Glucose formation decreased with increasing initial maltose concentration, suggesting inhibition of hydrolytic activity of this enzyme at high maltose concentration. Generally, TreS from thermophilic or mesophilic bacteria was used at reaction temperatures higher than 35°C. High amounts of glucose as a byproduct in the range of 7.3-19.2% were reported [16-18, 22, 32, 35, 37] due to increased flexibility of the protein structure at higher temperatures, making the catalytic pocket more accessible to water molecules that attacked the split glucose before the formation of α,α-1,1-glycosidic bonds [13, 49]. Our PmTreS showed an optimal reaction temperature of 40°C with lower glucose as a byproduct than previously reported, indicating its desirable properties and potential for future applications.

The effects of enzyme loading on trehalose yield and specificity were studied in reaction containing 100 g/l maltose using TreS loading of 1-4 mg/g substrate. The highest trehalose yield of 68.1% was obtained using an enzyme loading of 2 mg/g (Fig. 3B). With its high specific activity (90.2 U/mg protein) compared to TreS enzymes from other mesophilic bacteria (Table 1), this could allow the use of relatively low enzyme loading of PmTreS in the trehalose synthesis reaction.

### Optimization and Up-Scaling of Trehalose Production Reaction

We optimized the reactions in trehalose production using PmTreS by studying trehalose yield and specificity at various pH (7.0, 8.0, and 9.0) and temperature (30 and 40°C) in 50 ml reaction shaking flask experiments. The results revealed that the obtained trehalose yield was 64.0-65.7% at 24 h. Glucose formation (byproduct) ranged from 4.2-5.6% at 24 h, similar to that obtained in the small-scale experiments (Fig. S6). These findings suggested that high trehalose and low glucose formation could be obtained from PmTreS under these pH and temperature ranges.

**Fig. 4:**
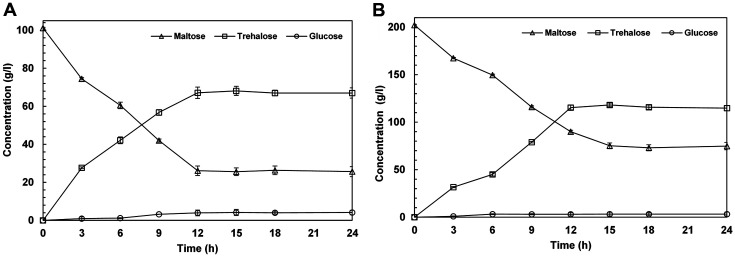
Up-scaled production of trehalose from 100 (**A**) and 200 (**B**) g/l maltose by PmTreS. The reactions were carried out in 50 mM glycine-NaOH buffer (pH 9.0) at 40°C for 24 h in a 2-L bioreactor (working volume, 1 L).

The trehalose production reaction was then studied in an up-scaled experiment in a 2-L bioreactor using 100 and 200 g/l maltose at pH 9, with PmTreS loading of 2 mg/g substrate. At 100 g/l maltose used as the substrate, the highest production of trehalose by PmTreS was 68.1 g/l at 15 h. (Fig. 4A). At 200 g/l, the trehalose concentration was 118.2 g/l at 15 h (Fig. 4B). A low concentration of glucose as a byproduct (~4 g/l) was obtained under the experimental conditions.

According to previous reports (Table 1), our trehalose yield was higher than that obtained using recombinant TreS from *D. radiodurans* (GenBank: AEJ36289), *A. aurescens* (GenBank: ACL80570.1) and *A. chlorophenolicus* (GenBank: AKA87409.1) [17, 23, 37] but slightly lower than TreS from *Pseudomonas* sp. (GenBank: AFK94626) and *P. stutzeri* (GenBank: AAF26837) [19, 35]. Compared to most reported mesophilic TreS enzymes, PmTreS showed desirable properties on yield and product specificity with lower byproduct yield than many previously reported enzymes under the experimental conditions, except for TreS from *P. stutzeri* (GenBank: AAF26837) [19]. However, PmTreS showed higher specific activity than the enzyme from *P. stutzeri*, potentially leading to lower enzyme cost and shorter reaction time and indicating the potential of the newly reported PmTreS for future applications.

Compared to the reported TreS from thermophilic microorganisms, the PmTreS-catalyzed reaction in our study achieved a higher trehalose yield and lower by-product formation in a shorter reaction time. The performance of TreS enzymes from *D. radiodurans* [17], *M. ruber* [22], and *P. torridus* [16] was demonstrated in trehalose production at 30-45°C and led to a trehalose yield of 56.0-61.0% with relatively higher glucose formation (7.8-9.2%) using a longer reaction time (24-72 h). The yield obtained in our study was only slightly lower than that from *Thermus antranikianii* [4] and *Thermobaculum terrenum* [40], which achieved more than 70% trehalose yield; however, their specificity as reflected by the formation of glucose byproduct was not reported for these enzymes. The alkalophilicity of PmTreS also potentially inhibits the growth of contaminated microorganisms in the production process [34]. This allows development of an energy-saving trehalose production process operated under mild condition.

In conclusion, the developed method for screening of TreS-producing strains was successfully demonstrated with advantages in terms of time, throughput, and scale. A new TreS enzyme from *P. montelii* showed desirable properties for trehalose production under mesophilic and alkaliphilic conditions, with lower byproduct formation compared to most previously reported enzymes. The mesophilic nature of the enzyme is desirable for its further application on cell surface display forms, either in bacteria or yeasts, in future development. Our demonstration of the enzyme-catalyzed trehalose production process in a bioreactor warranted the further study of this enzyme and showed it potential for future biotechnological application.

## Supplemental Materials

Supplementary data for this paper are available on-line only at http://jmb.or.kr.

## Figures and Tables

**Table 1:** Characteristics of recombinant trehalose synthases from mesophilic microorganisms.

Microorganism	Opt. pH	Opt. Temp (°C)	pH stability	Thermostability	Enzymatic conversion				

					Spec. activity * (U/mg)	Trehalose yield	Glucose yield (byproduct)	Condition Temp. | time	References
*Pseudomonas monteilii* TBRC 1196	9	40	>90% (pH 7-9, 12 h) ~61% (pH 10, 24 h)	>90% (20–40°C, 6 h) ~80% (20–40°C, 12 h) ~65% (20–40°C, 24 h) 14% (50°C, 2h)	90.6	68%	4%	40°C | 15 h	This study
*Pseudomonas* sp. [GenBank: AFK94626]	7.2	37	>95% (pH 7, 1 h) ~75% (pH 8, 1 h)	> 80% (10–40°C, 30 min) 10% (50°C, 30 min)	NR	70%	8%	37°C | 12 h	[35]
*Pseudomonas stutzeri* [GenBank: AAF26837]	8.5	35	pH 5.5–9.0	40% (55°C, 1 h)	79.2	75% 72%	0% 0%	15°C | 19 h 35°C | 19 h	[19]
*Pseudomonas putida* [GenBank: GU396283]	7.5	38	>95% (pH 7, 1 h) ~75% (pH 8, 1 h)	20% (42°C, 1h)	NR	72%	Not reported	38°C | NR	[36]
*Corynebacterium glutamicum* [GenBank: CAF20645]	7	35	~100% (pH 7.5, 1 h) ~70% (pH 8, 1 h)	80% (40°C, 30 min)	NR	67% 69%	~ 14% ~ 20%	25°C | 9 h 30°C | 9 h	[18]
*Deinococcus radiodurans*[GenBank: AEJ36289]	7.6	30	~100% (pH 7.5, 1 h) ~85% (pH 8, 1 h)	50% (40°C, 28.5 h) (50°C, 9.5 h)	11.4	58%	7.1%	30°C | 24 h	[17]
*Rhodococcus opacus* [GenBank: AGF84773]	7	25	~100% (pH 6.5–8.5, 1 h) ~80% (pH 10, 1 h)	~100% (15–45°C,1 h) 0% (60°C, 3 h)	NR	67%	12%	25°C | NR	[33]
*Arthrobacter aurescens*[GenBank: ACL80570.1]	6.5	35	~100% (pH 5.5–7.5, 20 min) ~70% (pH 8, 20 min)	~ 100% (20–35°C, 20 min) 10% (50°C, 20 min)	NR	59%	13%	30°C | 8 h	[37]
*Arthrobacter chlorophenolicus*[GenBank: AKA87409.1]	7.5	30	NR	40% (50°C, 6 h)	4.4	59%	17%	30°Cl 12 h	[23]

NR; not reported.

*One unit of enzyme activity was defined as the amount of enzyme that produced 1 μmol of trehalose from maltose per minute.

**Table 2:** Effects of metal ions on PmTreS activity.

Metal ions (1 mM)	Relative activity (%)
Zn^2+^	17.68 ± 0.00^b^
Mg^2+^	108.14 ± 1.64^f^
Ca^2+^	105.16 ± 2.58^ef^
Mn^2+^	106.85 ± 3.48^f^
Fe^2+^	100.88 ± 1.37^de^
Cu^2+^	1.84 ± 0.68^a^
Ba^2+^	96.51 ± 1.64^d^
Ni^2+^	27.42 ± 1.99^c^
Control	100.00 ± 1.64^d^

Different letters within the same column are statistically different at *p* ≤ 0.05.
