# Supplementary material for: Screening, Cloning, Expression and Characterization of New Alkaline Trehalose Synthase from Pseudomonas monteilii and Its Application for Trehalose Production
Source: J Microbiol Biotechnol. 2021 Aug 20;31(10):1455–64. doi: 10.4014/jmb.2106.06032 (PMC9705850; doi:10.4014/jmb.2106.06032)
Supplement: Supplementary file 1 [file jmb-31-10-1455-supple.pdf]

J. Microbiol. Biotechnol.
https://doi.org/10.4014/jmb.2106.06032

Supplementary Materials

Screening, cloning, expression and characterization of new alkaline trehalose synthase from *Pseudomonas monteilii* and its application for trehalose production

Srisakul Trakarnpaiboon[1], Benjarat Bunterngsook[1], Rungtiva Wansuksriand[2], Verawat Champreda[1*]

[1]*Enzyme Technology Team, Biorefinery and Bioproduct Technology Research Group, National Center for Genetic Engineering and Biotechnology, 113 Thailand Science Park, Paholyothin RD., Klong Luang District, Pathumthani, 12120, Thailand*

[2]*Cassava and starch Technology Research Team, Functional Ingredients and Food Innovation Research Group, National Center for Genetic Engineering and Biotechnology, Bangkok 10900, Thailand*

* Corresponding author. Tel.: +66 2564 6700 x 3446; fax**:** +66 2564 6707**.**

E-mail address**:** verawat@biotec.or.th (V. Champreda).

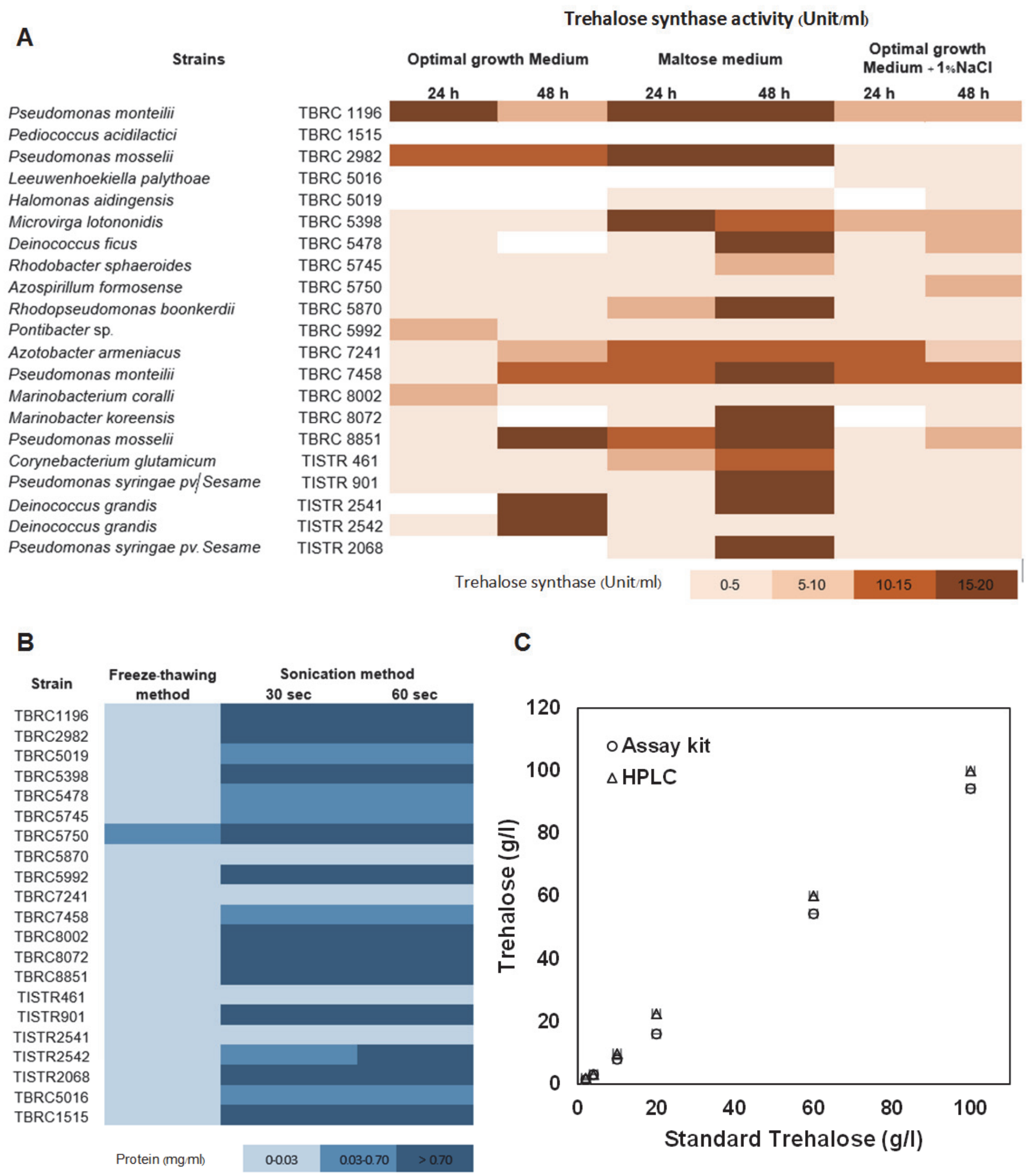

Fig. S1 (A) Trehalose synthase activity from diverse bacterial strains in different screening media cultivated for 24 and 48 hours. (B) Protein content of crude cell extract from different strains after freeze-thawing and sonication at 30 and 60 sec. (C) Correlation of two trehalose assay methods.

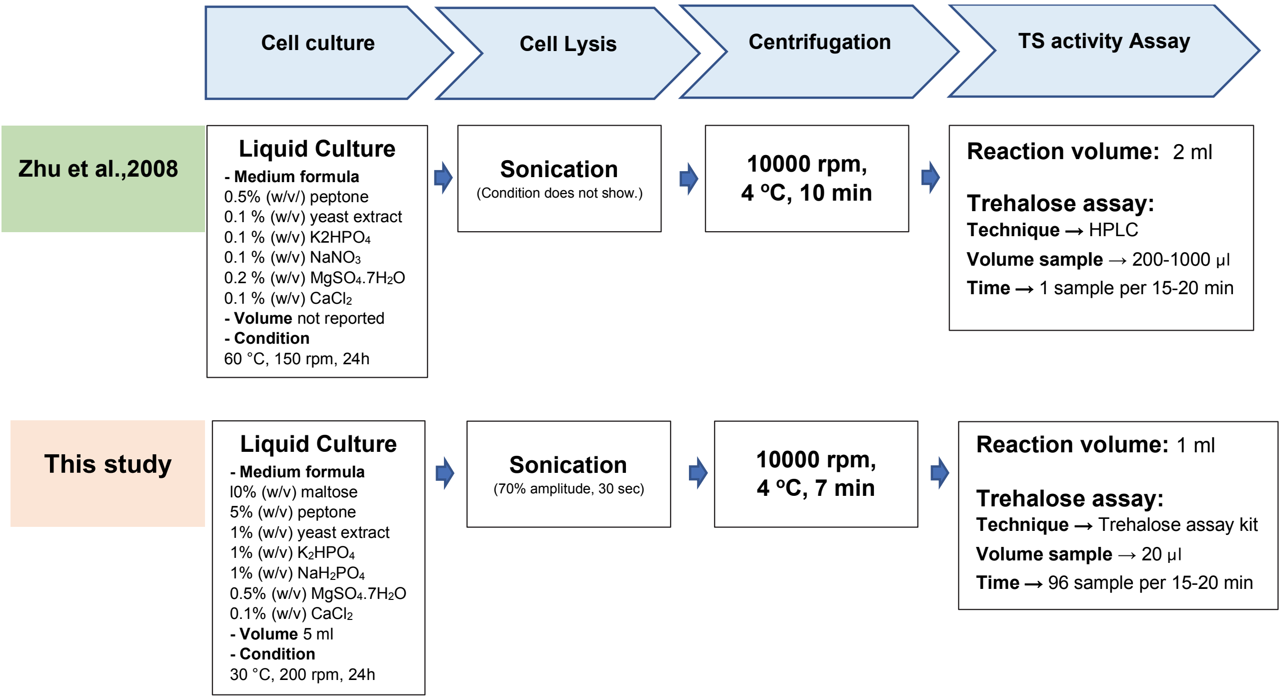

Fig.S2. Diagram of methods for screening of trehalose synthase-producing strains

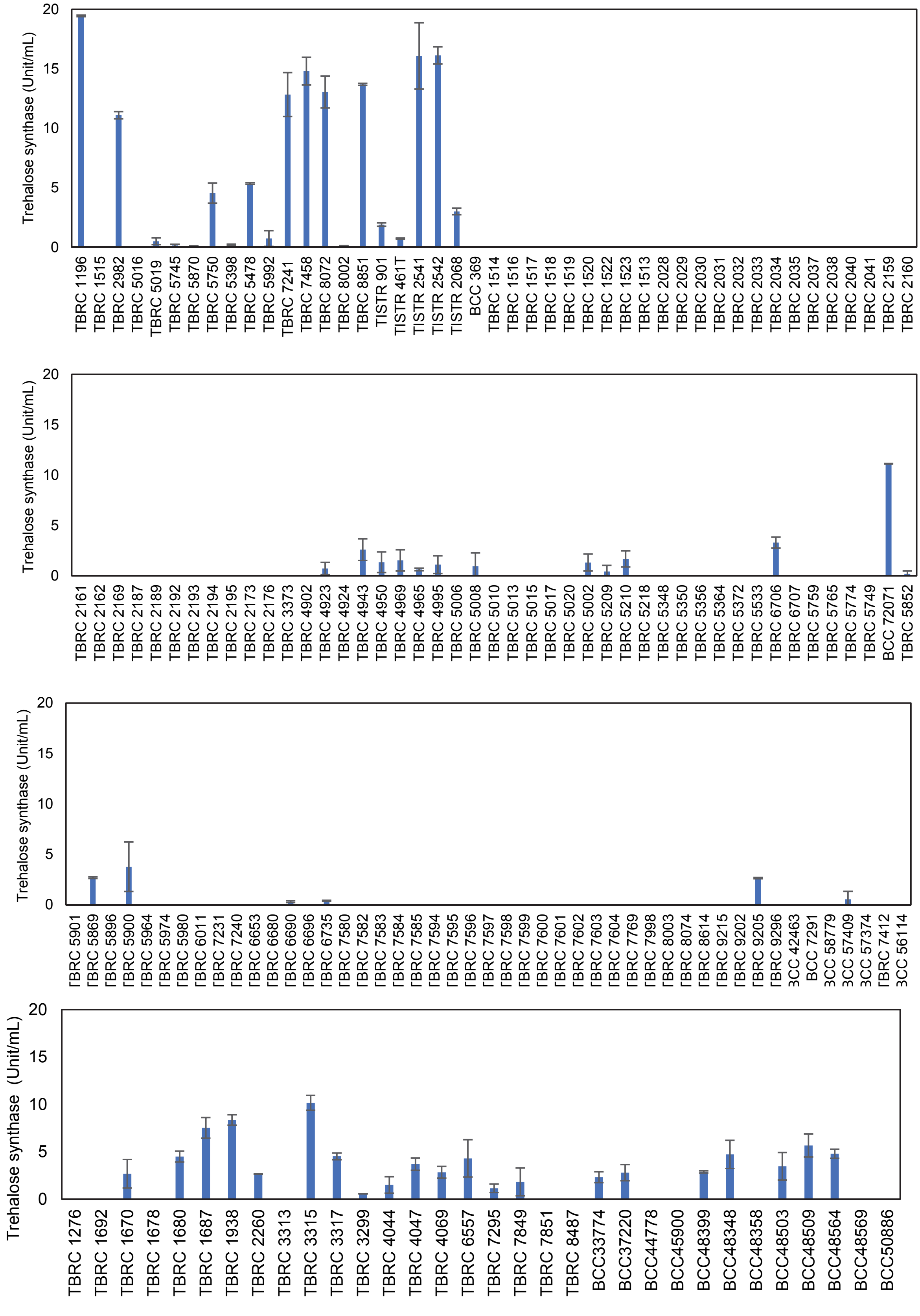

Fig.S3. Trehalose synthase activity of TBRC and TISTR strains in screening broth after incubation at 30°C for 24 hours.

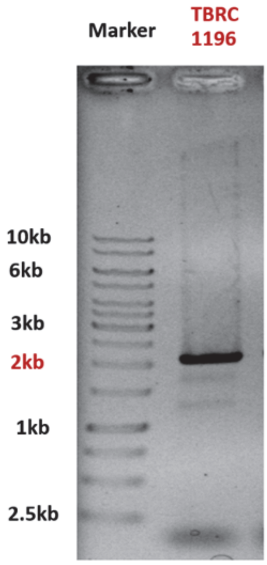

Fig.S4. Cloning of TreS gene from *P. monteilii* TBRC 1196. Marker: 1 kb DNA leader**.**

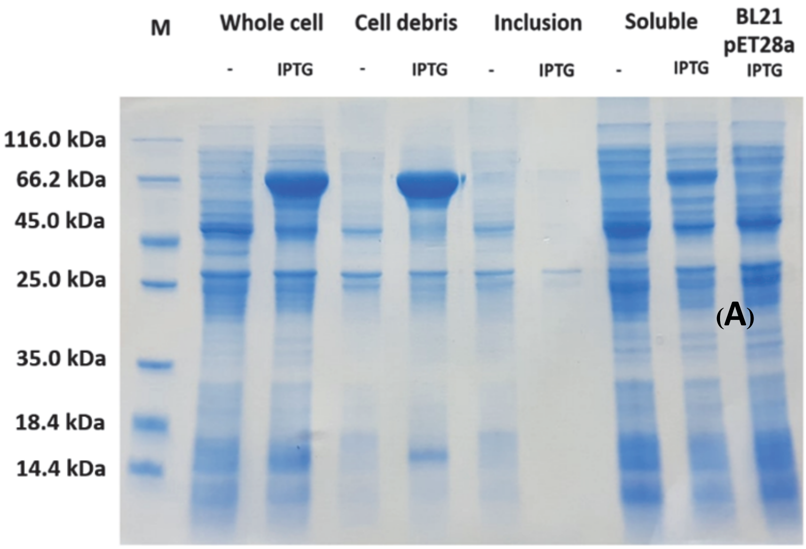

Fig.S5. SDS-PAGE analysis on protein profiles of the recombinant *E. coli* containing PmTreS gene. Lane M: standard protein marker. The recombinant clone was cultivated at 18°C and induced with 1 mM IPTG for 20 hours.

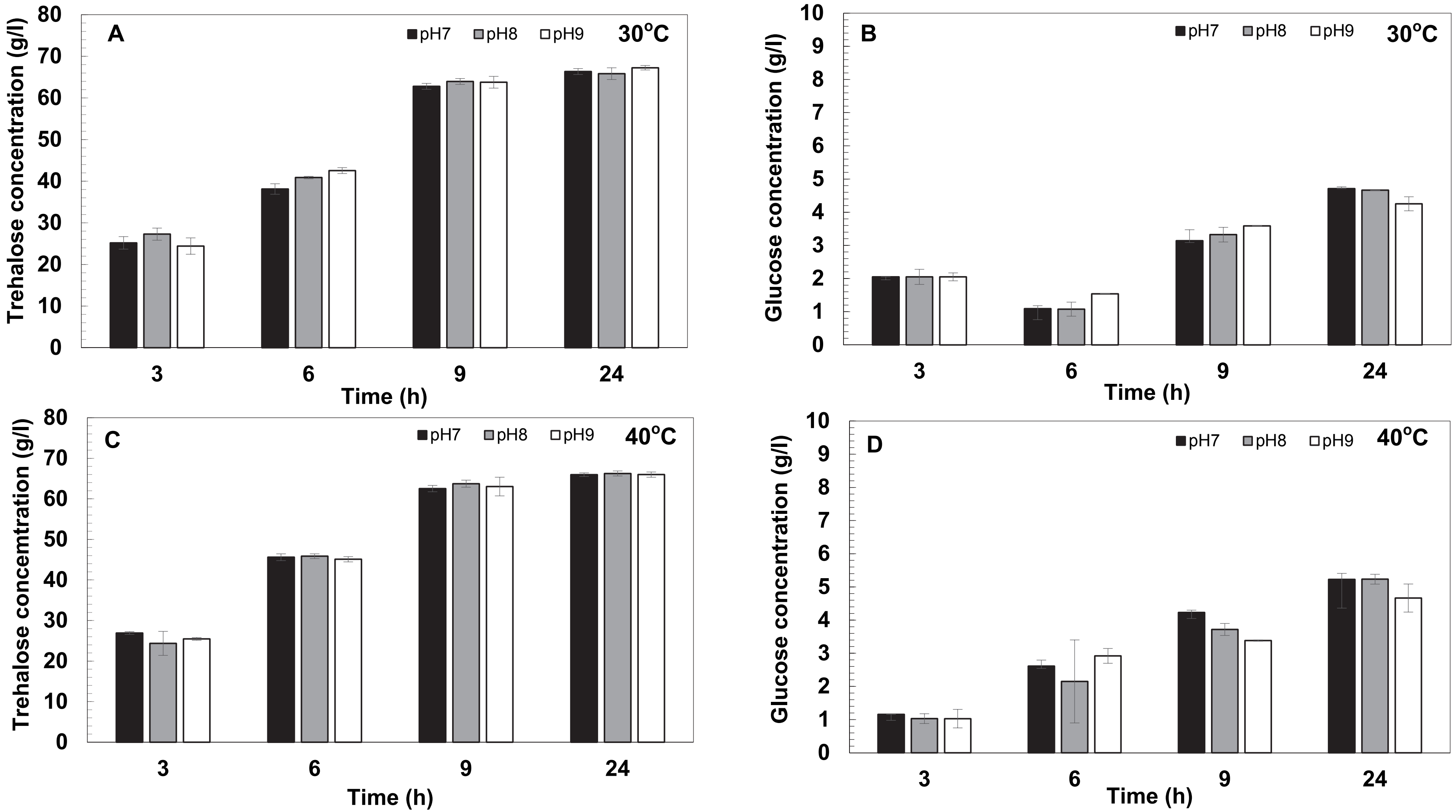

Fig. S6. Trehalose and glucose production by PmTreS at 30°C (A, B) and 40 °C (C, D). The reactions were carried out in 50 mM potassium phosphate buffer (pH 7.0 and 8.0) and 50 mM glycine-NaOH buffer (pH 9.0) with 100 g/l of maltose for 24 hours in a 250 ml shaking flask (working volume 50 ml).
